# Exploring the Association Between Digital Health Literacy and Burnout and Depression Among TV Journalists During the COVID-19 Pandemic in Serbia

**DOI:** 10.3390/healthcare13141688

**Published:** 2025-07-14

**Authors:** Ivana Bozovic, Aleksandra Jovic-Vranes, Ivana Stasevic-Karlicic, Dejana Stanisavljevic, Vedrana Pavlovic, Jovana Todorovic

**Affiliations:** 1 Serbian Broadcasting Corporation, Takovska 10, 11000 Belgrade, Serbia; ivanab.rts@gmail.com; 2 Institute of Social Medicine, Faculty of Medicine, University of Belgrade, Dr Subotica 15, 11000 Belgrade, Serbia; aleksandra.jovic-vranes@med.bg.ac.rs; 3 Clinic for Mental Disorders “Dr. Laza Lazarević”, Visegradska 26, 11000 Belgrade, Serbia; ivanastasevic73@gmail.com; 4 Faculty of Medicine, University of Priština—Kosovska Mitrovica, Anri Dinana, b.b., 38220 Kosovska Mitrovica, Serbia; 5 Ministry of Health Republic of Serbia, Nemanjina 22-26, 11000 Belgrade, Serbia; 6 Institute of Medical Statistics and Informatics, Faculty of Medicine, University of Belgrade, Dr Subotica 15, 11000 Belgrade, Serbia; dejana.stanisavljevic@med.bg.ac.rs (D.S.); vedrana.pavlovic@med.bg.ac.rs (V.P.)

**Keywords:** journalists, COVID-19, burnout, depression, health literacy

## Abstract

**Introduction:** As in other crises, during COVID-19 pandemic, journalists were under immense pressure to report precise scientific information in a timely manner, which may have had a negative influence on their mental health. There could be an association between the digital health literacy of journalists and their mental health. The aim of this article was to explore the association between digital health literacy and burnout and depression among journalists in Serbia. **Methods:** A cross-sectional study was conducted involving a total of 180 journalists working on television with national coverage in Serbia. The main research instrument used was a questionnaire with four sections containing personal demographic information, the Digital Health Literacy Instrument, the Maslach Burnout Inventory-Human Services Survey, and the Beck Depression Inventory. **Results:** A total of 30% participants were found to have high levels of burnout on the emotional exhaustion (EE) subscale. On the depersonalization (DP) subscale, 10.6% experienced high levels of burnout. On the personal accomplishment (PA) subscale, 38.3% of participants faced high levels of burnout. Multivariate logistic regression analyses showed the association between high burnout on the EE scale and health status (OR: 0.597, 95% CI: 0.375–0.952) and protecting privacy (OR: 0.522, 95% CI: 0.311–0.875). Multivariate logistic regression analysis showed the association between high burnout on the PA scale and information searching (OR: 0.255, 95% CI: 0.124–0.526), sex (OR: 2.594, 95% CI: 1.007–6.68), socioeconomic status (OR: 2.282, 95% CI: 1.133–4.595), and alcohol consumption (OR: 2.188, 95% CI: 1.004–4.769). Multivariate logistic regression analysis showed associations between depression and sex (OR: 0.180, 95% CI: 0.059–0.548), health status (OR: 0.316, 95% CI: 0.160–0.626), the use of anti-anxiety medications (OR: 7.303, 95% CI: 3.167–16.840), information searching (OR: 0.432, 95% CI: 0.191–0.981), and protecting privacy (OR: 0.443, 95% CI: 0.233–0.841). **Conclusions:** Our study showed a negative association between different domains of burnout, depression, and scores on protecting privacy and information searching scales.

## 1. Introduction

In late 2019, an unknown upper respiratory tract infection emerged in Wuhan, the capital of China’s Hubei province [1]. The disease spread rapidly worldwide. By early January 2020, it was determined that these infections were caused by a new strain of coronavirus, which had not been identified in humans until then. In February, the World Health Organization (WHO) named this disease COVID-19 [2]. The infection reached Serbia in March 2020, with the first case recorded in a traveler returning from Hungary [3].

In a response to the fast spread of the disease, quarantine measures were soon introduced in many European countries, and their citizens were confined to their homes and often required to work remotely. During this phase of the pandemic, online social networks became the primary source of information about the pandemic for a significant number of people, exposing them to a vast amount of different information, including misinformation and so-called ‘fake news’ [4]. There was the oversaturation of coverage, which made it difficult to distinguish between correct and incorrect information. As WHO Director-General Tedros Adhanom Ghebreyesus remarked at the 2020 Munich Security Conference: ‘We are not only fighting a pandemic; we are fighting an infodemic’ [5]. Research shows a high prevalence of mental health problems associated with frequent social media use during this time [6]. A study from Brazil found that television was the main source of the infodemic there, and the tendency to seek out information was higher among the elderly, people with lower incomes, and those who had frequent contact with infected individuals [7]. All of these groups exhibited increased feelings of fear, uncertainty, anxiety, depressive symptoms, and burnout syndrome at work [8].

Consequently, many countries reported high levels of anxiety, depression, and post-traumatic stress disorder among the general population. The main risk factors included being female, being of a younger age, having pre-existing psychiatric disorders or co-morbidities, and increased exposure to news on social networks [9].

Besides healthcare workers, journalists were also under immense pressure. They tracked local and global pandemic trends daily and intensified their efforts, with reporting on the pandemic resembling coverage of other crises [10]. The role of journalists is to provide the public with verified information and answer citizens’ questions, typically using a variety of sources (formal and informal, including personal contacts) [10]. The new assignments that arose during the pandemic were stressful for journalists, who are typically under pressure due to daily deadlines [11]. Additional stressors included changed working conditions, a shortage of human resources due to illness, having to report from hospitals and clinics, and conducting interviews with patients or their family members. Most journalists do not have a medical background, making unfamiliar terms, data, processes, and definitions further obstacles to accurate reporting [12]. In an international sample, 10% of journalists showed symptoms of post-traumatic stress disorder, while 26% exhibited symptoms of anxiety [12]. Another study indicated that 30% of journalists who frequently reported on the pandemic experienced higher levels of post-traumatic stress disorder symptoms [13]. Journalists also faced challenges in balancing remote work with family responsibilities, telecommuting, and managing news content remotely, all of which were linked to psychological stress [14].

Data from the literature indicate a possible association between the level of health literacy of journalists and their mental health, as they are often on the front line during emergencies, crises, and even catastrophic events [8]. The European Association for Health Literacy provided a definition, later adopted by the WHO in 2013: ‘Health literacy is related to literacy and implies the knowledge, motivation, and ability of individuals to access health information, understand it, evaluate it, and apply it to make informed decisions in everyday life regarding health, disease prevention, and improving health, to maintain the quality of life throughout the lifespan’ [15]. A satisfactory level of health literacy is essential for individuals to actively participate in making the right health decisions and engage in healthcare [16]. On the other hand, low health literacy can lead to problems in communication with healthcare workers, incorrect treatments, less frequent use of preventive services (such as screenings and immunizations), more frequent visits for examinations, longer hospital stays, increased mortality, and higher treatment costs [17]. As almost all health information in the past few decades has been provided online, digital spaces have become centers of communication, learning, and work but also places for prevention and health promotion. This has enabled the development of digital health literacy, which is defined as the ability to search for, find, understand, and evaluate health information from electronic sources and apply the acquired knowledge to approach or solve health problems, and its significance increased and was increasingly recognized during the COVID-19 pandemic [18].

To the best of our knowledge, no studies so far have examined burnout and depression among journalists in Serbia and its possible association with digital health literacy. The aim of this study was to examine the association of digital health literacy with burnout and depression among journalists in Serbia during the COVID-19 pandemic.

## 2. Materials and Methods

This was a cross-sectional study conducted among journalists from all television news programs (RTS, RTV, TV Pink, Happy TV, Prva TV, and TV B92) with national coverage. This study was conducted from March to December 2023. The sample size of 180 respondents was determined based on the prevalence of burnout in previous studies of about 3%, with a precision of 0.025 and a 95% confidence interval [19]. Permission was obtained from TV directors and/or the chief editors of news departments as appropriate. All participants received the information about this study, its processes, and aims and were asked to fill-in an online, anonymous questionnaire. Ethical approval was granted by the Ethical Review Board at the Medical Faculty, University of Belgrade (17/I-14). Participation was voluntary and anonymous; all the participants gave written consent for participation.

The primary research instrument was an online questionnaire, which consisted of four sections:

1. Personal demographic information. The first part of the questionnaire included the socio-demographic characteristics of the respondents (sex—male/female; age in years; marital status—married/single/separated/widowed; subjective socioeconomic status—very bad or bad/neutral/good/very good), lifestyle factors (e.g., alcohol consumption, use of anti-anxiety medications), and self-assessment of health status. In addition, data regarding COVID-19 infection and vaccination status were collected.
2. Digital Health Literacy Instrument (DHLI). A DHLI is a 21-item instrument that measures skills in seven categories: operational skills, navigation skills, and the participants’ ability to search for information, add self-generated content, evaluate reliability, determine the relevance of online information or any media information associated with COVID-19, and determine whether their online privacy is protected. In this study, the items were scored on a four-point Likert scale (from ‘very easy’ to ‘very difficult’, or from ‘never’ to ‘often’). Scores were then reversed, so that higher scores represented a higher level of digital health literacy, meaning that participants found it easy to search for health information, provide personal comments, determine whether the information was reliable, and apply information to their daily lives.

For the purpose of this study, we used five subscales to measure the participants’ ability to search for information, add self-generated content, evaluate reliability, determine relevance, and assess their online privacy. Each subscale consisted of 3 items, totaling 15 items. Further subscales for operational skills and navigation skills were not used in this study [18]. Cronbach’s alpha for the entire scale was α = 0.868; for the search for information subscale, it was α = 0.818; for the add self-generated content subscale, it was α = 0.836; for the evaluate reliability subscale, it was α = 0.770; for the determine the relevance subscale, it was α = 0.802; and for the assess the online privacy subscale, it was α = 0.490.

3. Maslach Burnout Inventory-Human Services Survey (MBI-HSS). The MBI-HSS was used to measure burnout. The questionnaire was used with permission from the copyright owner (Mind Garden, www.mindgarden.com (accessed on 11 December 2022)). The MBI-HSS is a 22-item scale and assesses three separate aspects of burnout: emotional exhaustion (EE; 9 items), depersonalization (DP; 5 items), and personal accomplishment (PA; 8 items). Responses were recorded on a 7-point Likert scale, ranging from 0, ‘never’, to 6, ‘every day’. Subscale scores were computed by adding item scores within the subscale and further classifying them as low, moderate, or high burnout according to established cut-off values (EE: 0–16, 17–26, ≥27; DP: 0–6, 7–12, ≥13; PA: 0–31, 32–38, ≥39). Higher values on the EE and DP subscales corresponded to a higher level of burnout, while higher values of PA indicated lesser degrees of burnout [20].
4. Beck’s Depression Inventory (BDI). BDI is the most commonly used instrument for the self-assessment of depressive symptoms. BDI can be used as a screening tool or as an instrument for the individual assessment of feelings and attitudes within the general problem of depression. This 21-item inventory, which takes approximately 10 min to complete, rates the intensity of symptoms on a four-point Likert scale from 0 (absence of symptoms) to 3 (severe symptoms). The total score, reflecting depression severity, ranges from 0 to 63, with higher scores denoting more severe depression. Interpretation varies by group: for individuals with psychiatric diagnoses, scores are segmented into normal/minimal (0–13), mild (14–19), moderate (20–28), and severe (29–63) depression. In contrast, for the general population, a score of 21 or higher suggests depression. Based on the score on BDI, the participants were classified as having depression (score of ≥21) or not having depression (score ˂ 21) [21].

The Maslach Burnout Inventory-Human Services Survey and Beck’s Depression Inventory have previously been used in Serbian, and we translated and back-translated the Digital Health Literacy Inventory in accordance with recommendations from the World Health Organization [22].

### Statistical Analysis

Descriptive statistics, including means and standard deviations for numerical variables and numbers and percentages for categorical variables, were used to characterize the study sample. Univariate and multivariate logistic regression analyses were used to determine independent predictors of burnout and depression after adjusting for age and gender. MBI-HSS subscales and BDI were used as dependent variables in separate regression models. Multivariate regressions were conducted in a stepwise manner (forward Wald) using the Ordinary Least Squares method. Results were expressed as B, Wald Chi-Square, odds ratios (ORs), and their 95% confidence intervals (CIs). Variables were included in the multivariate regression analysis if they were significant at the *p* < 0.10 level according to the results of the univariate analysis. For the implementation of regression analysis, model assumptions were taken into account. All tests were two-tailed. *p* < 0.05 was considered statistically significant. Statistical analysis was conducted using IBM SPSS Statistics 25 software.

## 3. Results

The DHLI, the MBI-HSS, and BDI were completed by 180 television news journalists. Most of the participants were female, accounting for 78.3% of the study sample. The average age was 45.6 (SD 10.2), with the youngest participants aged 25 and the oldest aged 63 years old. Among the participants, 61.1% were married, while 28.9% were single. Subjective socioeconomic status was categorized into four groups: very bad and bad, neutral, good, and very good. The largest group of participants (52.8%) was in the neutral category, followed by 37.2% with good socioeconomic status. A small percentage, comprising 1.1% of the sample, or two individuals, was classified as having very bad or bad socioeconomic status. All the demographic characteristics of the study population are presented in Table 1.

In the study population, the distribution of health status was as follows: 1.1% of respondents reported their health status as very bad or bad, 38.3% as neutral, and almost half of the participants (48.9%) reported their health status as good. Regarding the use of anti-anxiety medications, 31.1% of the participants confirmed usage, while alcohol consumption was reported by 75% of the participants. Vaccine uptake was high, with 83.9% of the population being vaccinated. Lastly, 34.4% of the study population reported a score indicating depressive symptoms (Table 2).

The frequencies of responses to DHLI items are presented in Table 3. When searching for COVID-19 information online, a majority of participants found it easy or very easy to make choices from the information that they encountered (69.4% found it easy, 17.8% very easy) and to use appropriate search terms to find what they were looking for (65.6% easy and 29.4% very easy). Additionally, 60% of participants found it easy to locate the exact information that they needed.

In terms of adding self-generated content, a majority expressed ease in clearly formulating health-related questions or concerns (67.8% easy, 22.8% very easy). Similarly, most participants felt confident in writing messages that others could understand (66.7% easy, 18.3% very easy). Evaluating the reliability of information posed more of a challenge, with 45.6% finding it difficult to ascertain the reliability of COVID-19 information and 44.4% finding it easy. The task of discerning commercial interests in the information was also challenging, with 60.6% finding it easy and 22.2% finding it difficult.

When determining the relevance of information, a majority of respondents were able to decide if the information applied to them (61.7% easy and 13.9% very easy) and could apply it in their daily lives (63.3% easy and 12.2% very easy). Furthermore, 68.9% found it easy, and 16.7% very easy, to use the information for making health-related decisions.

In terms of protecting their online privacy, while a considerable number of participants were confident in managing their privacy when posting on public forums or social media (39.4% very easy), a notable percentage found it challenging to judge who could read their posts (20.6% very difficult) (Table 3).

The distribution of participants with low, moderate, and high levels of burnout, along with mean scores and standard deviations for each burnout subscale, are presented in Table 4. For the EE, the mean score was 19.6 (SD 12.9). The distribution indicates that almost half, i.e., 48.9% of participants, experienced low levels of burnout; 21.1% were categorized as experiencing moderate burnout; and 30% were found to have high levels of burnout. On the DP subscale, the mean score was 4.9 (SD 5.3). A significant majority (73.3%) reported low burnout levels, whereas 16.1% and 10.6% experienced moderate and high levels of burnout, respectively. On the PA subscale, a substantial 38.3% of individuals faced high levels of burnout (Table 4). A total of 16 (8.9%) participants had high burnout on both the EE and DP scales.

A multivariate logistic regression analysis was conducted to identify independent factors associated with the burnout subscales EE, DP, and PA. The findings are presented in Table 5. For the EE, the subscales for perceived health status (*p* = 0.03) and online privacy protection (*p* = 0.01) were significant predictors of burnout after adjusting for age and gender. Journalists with lower scores on the protecting privacy subscale and with worse health statuses were more prone to EE.

Within the DP subscale, vaccination status (*p* = 0.02) stood out as a significant factor, indicating that journalists who were not vaccinated were more likely to experience depersonalization. For the PA, several variables were identified as significant: sex (*p* = 0.05), socioeconomic status (*p* = 0.02), alcohol consumption (*p* = 0.049), and information searching (*p* < 0.001). Women exhibited a higher likelihood of experiencing moderate-to-high burnout. Additionally, a higher subjective socioeconomic status, alcohol consumption, and a lower score on the information searching subscale were found to be independent factors associated with burnout among journalists.

In a multivariate logistic regression analysis examining factors associated with depression, the model identified several independent factors associated with depression. Men showed a lower risk of depression compared with women, journalists with better health status demonstrated a decreased likelihood of depression, and journalists who used anti-anxiety medications were found to have a higher risk of experiencing depression (*p* = 0.003, *p* = 0.001, and *p* < 0.001, respectively). Lower scores on the information-searching subscale were linked to a higher risk of depression (*p* = 0.04). Additionally, journalists with lower scores on the protection of privacy subscale tended to have a higher risk of depression (*p* = 0.01) (Table 6).

## 4. Discussion

In this study, our aim was to examine the prevalence of high burnout and depression among journalists in Serbia during the COVID-19 pandemic, as well as its association with digital health literacy. The prevalence of high burnout on the EE scale in our study was 30%, the prevalence of high burnout on the depersonalization scale was 10.6%, and the prevalence of high burnout on the low personal accomplishment scale was 38.3%. Burnout on the EE scale was negatively associated with health status and score in the protecting privacy category of the DHLI, high burnout on the depersonalization scale was negatively associated with vaccination, and high burnout on the low personal accomplishment scale was positively associated with female sex, alcohol consumption, and socioeconomic status and negatively associated with score in the information searching category of the DHLI. Depression in our study was negatively associated with female sex, health status, and scores in the information searching and protecting privacy categories of the DHLI and positively associated with use of anti-anxiety medications.

This study was conducted three years after the start of the pandemic, from March to December of 2023, and burnout prevalence was relatively high among journalists on each of the examined subscales. This suggests that despite a certain degree of adaptation to the pandemic, as well as numerous systemic measures implemented against it (such as vaccination, more COVID-19 testing resources, and social contact management) [23], the psychological strain on respondents was still notable.

Journalists likely faced challenges in reporting from hospitals and conducting interviews with doctors and nurses or patients and their families, which may have contributed to the psychological stress that they experienced [24]. One study has shown that journalists who were repeatedly covering COVID-19 stories had significantly higher levels of psychological distress and PTSD symptoms—but not depression—than journalists who worked during the pandemic but did not report on it [13]. In another study, more than 80% of journalists in a sample showed evidence of clinically significant emotional distress [9]. Although these studies have variable results, they all indicate the psychological strain that the journalists were exposed to. These findings are supported by our own study.

Almost 80% of our sample were women, which is in line with the existing data on sex distribution among journalists in Western Balkan countries, where the majority of journalists are female, and data from Croatia that state that almost 80% of students in journalism are female [25].

In our research, better health status was associated with an almost 40% lower likelihood of developing burnout on the emotional exhaustion subscale. One study that lasted from 2018 to 2022 shows that at the end of that period, healthcare workers experienced a decrease in burnout if they trusted management, had enough time to complete work, had help from supervisors, and felt that their workplace supported productivity [24].

Journalists in our study who were better at protecting their online privacy had an almost 50% lower likelihood of developing burnout on an emotional exhaustion subscale. Those who had been vaccinated were almost three times less likely to develop burnout, as measured on a depersonalization scale. More than 80% of our sample was vaccinated against COVID-19, which is more than in the majority of the studies examining COVID-19 vaccination in the general population, or even in healthcare professionals such as nurses [26].

Many journalists may have recognized the importance of being vaccinated as they considered themselves role models for the general population. The association between vaccination status and burnout has not been thoroughly examined; however, one study in Greece has shown that COVID-19-related burnout was associated with a lower vaccination rate [26].

In our study, women were 2.59 times more likely to develop burnout as measured on the low personal accomplishment subscale. This has been shown in previous research [27]. A higher self-assessed socioeconomic status was associated with more than twice the likelihood for burnout on the low personal accomplishment subscale in our study. This finding is contrary to previous studies in which the stress associated with lower socioeconomic status was recognized as a factor for the development of burnout [28].

Alcohol consumption was associated with a greater likelihood of burnout in our study. Another study has shown that those who were more affected by the COVID-19 pandemic tended to consume more alcohol (more days, more drinks). The reasons for this may include increased stress due to the pandemic, the availability of alcohol, and boredom [29].

Those who rated their ability to search for information as ‘good’ had a four times lower likelihood of developing burnout. Journalists who assessed performing an information search on COVID-19 as ‘easy’ or ‘very easy’ had an almost four times lower likelihood of having a score indicative of high burnout on the low personal accomplishment subscale. Higher self-confidence in accessing the necessary information may significantly increase the sense of control over one’s work, allow for the capacity to influence it, enable access to important resources, and, finally, allow a journalist to be more effective in their job, all of which can decrease the likelihood of burnout [30].

In our study, women were five times less likely to develop depression, while, in addition, those with better health status were three times less likely to develop depression. Those who used anti-anxiety medications had a 7.3 times higher probability of developing depression. In addition, those who scored lower on information searching had a greater likelihood of depression, suggesting that an ease in acquiring information may promote mental health. Also, a higher risk of depression was associated with lower scores for the protection of privacy, which may correspond with increasing worries about online privacy, information security, and privacy protections [31].

The main limitation of this study is its cross-sectional design, as it does not allow for the establishment of causal relationships between the variables. Another is that this study included only TV journalists from television programs with national coverage, and the results could not be generalized to the entire population of journalists in Serbia. However, the main strength of our study is that, to the best of our knowledge, it is the first study that examines both digital health literacy and mental health among journalists in one country in South-Eastern Europe.

## 5. Conclusions

In summary, our study shows that journalists who covered the COVID-19 pandemic experienced similar levels of burnout and depression to those on the front lines of the fight against the virus. Burnout was associated with the information searching and protecting privacy categories of digital health literacy. High burnout on the emotional exhaustion scale was associated with a lower score in protecting privacy category and with a worse health status. High burnout on the low personal accomplishment scale was associated with a lower score in the information searching category of digital health literacy. Depression was also associated with lower scores in the protecting privacy and information searching categories. Promoting digital health literacy and the skills that journalists use on a daily basis when reporting on health and healthcare topics can have a significant impact on their well-being and efficiency at work during crisis situations such as a pandemic. There is a clear need for the additional training of journalists on medical topics.

## Figures and Tables

**Table 1:** The demographic characteristics of the study population.

Variable	N (%)
Sex	
Female	141 (78.3%)
Male	39 (21.7%)
Age *, years	45.6 ± 10.2
Marital status	
Married	110 (61.1%)
Single	52 (28.9%)
Separated	16 (8.9%)
Widowed	2 (1.1%)
Subjective Socioeconomic status	
Very bad, bad	2 (1.1%)
Neutral	95 (52.8%)
Good	67 (37.2%)
Very good	16 (8.9%)

* Data are presented as mean ± (SD).

**Table 2:** The health-related characteristics of the study population.

Variable	N (%)
Self-reported health status	
Very bad, bad	2 (1.1%)
Neutral	69 (38.3%)
Good	88 (48.9%)
Very good	21 (11.7%)
Use of sedatives	
Yes	56 (31.1%)
No	124 (68.9%)
Vaccine	
Yes	151 (83.9%)
No	29 (16.1%)
Alcohol usage	
Yes	135 (75.0%)
No	45 (25.0%)
Depression	
Yes	62 (34.4%)
No	118 (65.6%)

**Table 3:** Participants’ frequency of responses to DHLI items.

Items	Very Difficult	Difficult	Easy	Very Easy	Mean (SD)
**INFORMATION SEARCHING** ** When you search the Internet for information on COVID-19, how easy or difficult is it for you to…**					3.1 (0.5)
Make a choice from all the information you find?	2 (1.1%)	21 (11.7%)	125 (69.4%)	32 (17.8%)	
Use the proper words or search query to find the information you are looking for?	1 (0.6%)	8 (4.4%)	118 (65.6%)	53 (29.4%)	
Find the exact information you are looking for?	2 (1.1%)	36 (20%)	108 (60.0%)	34 (18.9%)	
**ADDING SELF-GENERATED CONTENT** ** When typing a COVID-19-related message (e.g., to your doctor, on a forum, or on a social media platform such as Facebook or Twitter), how easy or difficult is it for you to…**					3.1 (0.5)
Clearly formulate your question or health-related worry?	0 (0)	17 (9.4%)	122 (67.8%)	41 (22.8%)	
Express your opinion, thoughts, or feelings in writing?	1 (0.6%)	32 (17.8%)	110 (61.1%)	37 (20.6%)	
Write your message as such that people understand exactly what you mean?	1 (0.6%)	26 (14.4%)	120 (66.7%)	33 (18.3%)	
**EVALUATING RELIABILITY** ** When you search the Internet for information on COVID-19, how easy or difficult is it for you to…**					2.8 (0.5)
Decide whether the information is reliable or not?	7 (3.9%)	82 (45.6%)	80 (44.4%)	11 (6.1%)	
Decide whether the information is written with commercial interests (e.g., by people trying to sell a product)?	4 (2.2%)	40 (22.2%)	109 (60.6%)	27 (15.0%)	
Check different websites to see whether they provide the same information?	1 (0.6%)	30 (16.7%)	120 (66.7%)	29 (16.1%)	
**DETERMINING RELEVANCE** ** When you search the Internet for information on COVID-19, how easy or difficult is it for you to…**					2.9 (0.5)
Decide if the information you found is applicable to you?	1 (0.6%)	43 (23.9%)	111 (61.7%)	25 (13.9%)	
Apply the information you found in your daily life?	0 (0)	44 (24.4%)	114 (63.3%)	22 (12.2%)	
Use the information you found to make decisions about your health (e.g., preventive measures, maintaining hygiene, transmission routes and risk prevention)?	3 (1.7%)	23 (12.8%)	124 (68.9%)	30 (16.7%)	
**PROTECTING PRIVACY** ** When you post a COVID-19-related message on a public forum or social media, how often…**					3.4 (0.6)
	**Never**	**Once**	**Few times**	**Often**	
Do you find it difficult to judge who can read along?	37 (20.6%)	54 (30.0%)	18 (10.0%)	71 (39.4%)	
Do you (intentionally or unintentionally) share your own private information (e.g., name or address)?	3 (1.7%)	25 (13.9%)	9 (5.0%)	143 (79.4%)	
Do you (intentionally or unintentionally) share some else’s private information?	2 (1.1%)	6 (3.3%)	3 (1.7%)	169 (93.9%)	

**Table 4:** Burnout scores among journalists.

Domain	Mean (SD)	Low Burnout	Moderate Burnout	High Burnout
EE	19.6 (12.9)	88 (48.9%)	38 (21.1%)	54 (30%)
DP	4.9 (5.3)	132 (73.3%)	29 (16.1%)	19 (10.6%)
PA **	34.0 (8.8)	61 (33.9%)	50 (27.8%)	69 (38.3%)

Notes: Cutoffs for low/moderate/high burnout are as follows: EE: 0–16, low; 17–26, moderate; ≥27 high. DP: ≥13, high; 7–12, moderate; 0–6, low. PA: ≥39, low; 32–38, moderate; 0–31 high. ** The PA subscale is interpreted in the opposite direction to the EE and DP subscales.

**Table 5:** Multivariate logistic regression models with MBI-HSS subscales as dependent variables.

Variables	*B*	Wald Chi-Square	*p*	OR	95% CI for OR	
					Lower	Upper
EE						
Health status	−0.516	4.700	0.03	0.597	0.375	0.952
Protecting privacy	−0.650	6.081	0.01	0.522	0.311	0.875
DP						
Vaccination status	−0.991	5.556	0.02	0.371	0.163	0.846
PA						
Sex	0.953	3.899	0.05	2.594	1.007	6.683
Subjective Socioeconomic status	0.825	5.338	0.02	2.282	1.133	4.595
Alcohol consumption	0.783	3.881	0.049	2.188	1.004	4.769
Information searching	−1.367	13.664	<0.001	0.255	0.124	0.526

OR, odds ratio; 95% CI, 95% confidence interval.

**Table 6:** Multivariate logistic regression model with depression as dependent variable.

Variables	*B*	Wald Chi-Square	*p*	OR	95% CI for OR	
					Lower	Upper
Sex	−1.715	9.125	0.003	0.180	0.059	0.548
Health status	−1.152	10.929	0.001	0.316	0.160	0.626
Use of anti-anxiety medications	1.988	21.756	<0.001	7.303	3.167	16.840
Information searching	−0.839	4.027	0.04	0.432	0.191	0.981
Protecting privacy	−0.815	6.200	0.01	0.443	0.233	0.841

OR, odds ratio; 95% CI, 95% confidence interval.

## Data Availability

The data can be made available upon a reasonable request to the corresponding author.

## Abbreviations

The following abbreviations are used in this manuscript:

DHLI	Digital Health Literacy Instrument
MBI-HSS	Maslach Burnout Inventory-Human Services Survey
BDI	Beck’s Depression Inventor
WHO	World Health Organization
EE	Emotional Exhaustion
DP	Depersonalization
PA	Personal Accomplishment
SD	Standard Deviation
CI	Confidence Interval
OR	Odds Ratio
